# Basigin-mediated redistribution of CD98 promotes cell spreading and tumorigenicity in hepatocellular carcinoma

**DOI:** 10.1186/s13046-015-0226-6

**Published:** 2015-10-06

**Authors:** Bo Wu, Yi Wang, Xiang-Min Yang, Bao-Qing Xu, Fei Feng, Bin Wang, Qiang Liang, Yu Li, Yang Zhou, Jian-Li Jiang, Zhi-Nan Chen

**Affiliations:** Cell Engineering Research Centre & Department of Cell Biology, State Key Laboratory of Cancer Biology, Fourth Military Medical University, 169 Changle West Road, Xi’an, 710032 P. R. China

**Keywords:** Hepatocellular carcinoma, Basigin, CD98, Distribution, Endosomes, Progression

## Abstract

**Background:**

Dysregulated endocytosis of membrane proteins contributes significantly to several hallmarks of cancer. Basigin can enhance cancer progression, but its precise mechanism remains unclear. CD98 promotes cell spreading and tumorigenicity by triggering integrin clustering and enhancing cell adhesion to the extracellular matrix. The endocytosis and recyle of basigin and CD98 might play critical roles in cancer.

**Methods:**

The role of CD98 was confirmed in liver cancer cells by cell spreading *in vitro* and tumorigenicity by nude mice xenograft tumor assay *in vivo*; membrane expression of basigin and CD98 in SMMC-7721 was measured by FCAS; pull down and SPR analysis were uses to reveal the direct association between basigin and CD98; DsRed1 tagged CD98 was blocked in the cytoplasm in K7721 (whose basigin was knockn out) and had a well colocalization with ER and Rab5a positive recycling endosomes under co-focal; finally, by FRET imaging and FCAS we observed the internalization of basigin and CD98 was flotillin-1-regulated, and their recycle at early steps was Arf6-mediated.

**Results:**

Basigin and CD98 were highly expressed and co-localized on the human hepatocellular carcinoma (HCC) cell membrane; basigin can directly bind to CD98, mediating CD98 redistribution on the HCC cell membrane and activating the downstream integrin signaling pathway. Internalization of basigin and CD98 was flotillin-1 regulated the and their recycling was mediated by Arf6. This recycling process for basigin and CD98 promotes cell spreading and tumor growth in liver cancer xenografts.

**Conclusion:**

Basigin, as a redistribution chaperone of CD98, plays a critical role in promoting cell spreading and the progression of hepatocellular carcinoma.

**Electronic supplementary material:**

The online version of this article (doi:10.1186/s13046-015-0226-6) contains supplementary material, which is available to authorized users.

## Background

The aberrant distribution of membrane proteins, including immune adhesion molecules, conjunction proteins and many RTKs (receptor tyrosine kinases), can create markedly altered tissue polarity and instigate motile phenotypes and cancerous cell behavior in normal cells [1–3].

Dysregulated endocytosis contributes significantly to several hallmarks of cancer [4]. Membrane proteins could be internalized through CME (clathrin-mediated endocytosis) or CIE (clathrin-independent endocytosis). Clathrin, adaptor proteins, dynamin and other GTPases participate in and regulate the process of CME [5]. Caveolin, flotillin-1, RhoA and Arf6 were reported to mediate different types of CIE [6, 7]. After endocytosis, the internalized proteins (both CME and CIE protein) first converge into Rab5a-positive endosomes. Then, a series of sorting events affect the fate of the internalized proteins, resulting in either degradation in the lysosomes or recycling back to the plasma membrane [8–11]. The recycling endosomes control the amplitude and duration of oncogenic signaling from cancerous cells by regulating receptor recycling and redistribution [4, 12]. Although the basic features of the endocytic process had been established more than two decades ago, researches on how the vesicles are recycled to mediate the distribution of tumor drivers, especially the CIE proteins, are rare.

HCC is a common malignancy worldwide, and to develop targeted diagnostic tools and novel therapies efforts have been tried to understand the molecular basis of HCC [13–15]. Basigin, also known as CD147 or EMMPRIN (extracellular matrix metalloproteinase inducer), is gradually being recognized as a cancer-associated biomarker, and significant clinical therapeutic progress has been made using anti-basigin monoclonal antibodies that target HCC [16, 17]. Previously, we identified the downstream effectors of basigin, such as PI3K, TGF-β and MMPs, which can promote liver cancer phenotypes and behavior [18–20], but the precise mechanism remains obscure. Identifying the binding partners of basigin could help elucidate how basigin functions in cancer progression. CD98 was reported to be associated with basigin [21, 22], contributing to tumor growth in a variety of cancers with a high expression level of this protein [23–25]. CD98 can promote the activation of β1 and β3 integrins by FAK phosphorylation, which eventually promotes cancer cell survival, proliferation and migration [26, 27]. As these two important tumor-associated membrane proteins are both internalized through CIE [28], further clarifying how basigin and CD98 participate in and undergo vesicle recycling is critical to understand the behaviors and malignant phenotypes of cancer cells.

In this work, we confirmed that the redistribution of CD98 is mediated by direct interaction with basigin and that they promote the malignant phenotypes of HCC cells through flotillin-1-mediated internalization and the early steps of Arf6-mediated recycling.

## Materials and methods

### Cell culture and reagents

Human SMMC-7721 HCC cells (Institute of Biochemistry and Cell Biology, Academic Sinica, Shanghai, China) were cultured in RPMI1640 (Gibco, New York, USA) with 10 % fetal bovine serum, 2 mM glutamine, 100 U/ml penicillin, and 100 μg/ml streptomycin in 5 % CO_2_ atmosphere at 37 °C. K7721 cells (developed from SMMC-7721 cells by a zinc-finger nuclease-targeted knockout of the basigin gene [29]) were cultured in the same conditions. The human liver carcinoma cells HepG2 and Huh7 were obtained from ATCC were cultured in DMEM medium (Gibco, New York, USA) containing 10 % fetal bovine serum.

The CD98 antibody (for immunofluorescence) was purchased from Abcam (ab23495), rabbit polyclonal antibodies to CD98 (for western blot) were from Wuhan Co., China (15193-1-AP), and rabbit polyclonal antibodies to CD147 (sc-13976) and Flotillin-1(sc-74566) were from Santa Cruz Biotechnology. Antibodies to β1 integrin and activated β1 integrin were from Abcam (ab3167) and Millipore Co. (mab2079Z), respectively. Antibodies to CD98-PE (556077) and CD147-FITC (555962) for flow cytometry were from BD Bioscience. Rabbit polyclonal antibodies to Akt and p-Akt were from CST (9272S) and Abcam (ab81283), respectively. Antibodies to basigin (HAb18, IgG1) and α-tubulin were developed in our laboratory.

### Plasmids

The following plasmids were used: pET32a(+) (Invitrogen, Carlsbad, CA, USA), peGFP-N1 (Clontech, Mountain View, CA, US), pcDNA3.1 (Invitrogen, Carlsbad, CA, USA), pcDNA3.1-CD147 (NM_198589), pdsRed1-N1-CD147 (CD147-dsRed1) and pCMV5-CD98 (NM_002394.5). The CD98 sequence coding for the CD98-ED (residues 111–530 of CD98) was cloned into the pET32a(+) plasmid with *Nde* I/*Xho* I. Sequences encoding eGFP were inserted into the signal peptide and coding sequense of CD147 by overlapping PCR (Polymerase Chain Reaction) and then cloned into peGFP-N1 with Hind III/Not I to create peGFP-CD147(N). Then the intracellular or extracellular domain of CD147 were deleted to generate peGFP-CD147(N)-out and peGFP-CD147(N)-in, respectively. Similarly, the coding sequences of CD98, along with DsRed1 from pDsRed1-N1, were cloned into pcDNA3.1 with *Nhe* I/*Xho* I to generate pcDNA3.1-dsRED1-CD98. Wild-type (accession no. NM_001663.3) and mutated Arf6 (Q67L) were cloned into pcDNA3.1 with *Nhe* I/*Xho* I to generate pcDNA3.1-Arf6 and pcDNA3.1-Arf6Q67L respectively. Flotillin-1(NM_005803.2) were cloned and inserted into peGFP-N1 to create Flotillin-1-eGFP.

Validated expression plasmids for pLKO–SLC3A2(CD98) shRNA (CCGGCGAGAAGAATGGTCTGGTGAACTCGAGTTCACCAGACCATTCTTCTCGTTTTTG) and pLKO–control shRNA plasmid were purchased from Sigma. EGFP-Rab5AQ79L was a gift from Dr. Qing Zhong (University of California, Berkeley) and shared through Addgene (Plasmid 28046). GFP-Rab11 was from Dr. Richard Pagano (Mayo Foundation for Medical Education and Research) via Addgene (Plasmid 12674). The QuikChange Lightning Multi Site-Directed Mutagenesis Kit was from Stratagene, Santa Clara, CA, USA. The complete sequence of the above constructs can be obtained upon request.

### RNA interference and transfection

Small interfering RNA (siRNA) sequences for CD147-siRNA: GTACAAGATCACTGACTCT and CD98-siRNA: GAGCCGAGAAGAAUGGUCUGGUGAA were designed and synthesized by Shanghai GenePharma (Shanghai, China). RNA oligonucleotides to the conserved regions in human flotillin-1 (UGAGGCCAUGGUGGUCUCCdTdT and CACACUGACCCUCAAUGUCdTdT) were prepared as duplexes with overhanging dTs (GenePharma, Shanghai, China) [6]. The cells were transfected with the siRNAs or plasmids using Lipofectamine 2000 (Invitrogen, Carlsbad, CA, US). siGAPDH (5’-GUA UGA CAA CAG CCU CAA GTT-3’) and snc-RNA (5’-UUC UCC GAA CGU GUC ACG UTT-3’) were used as positive and negative controls, respectively, under similar conditions.

### Quantitative real-time PCR (qRT-PCR)

Total RNA was extracted using an Omega R6934-01 Total RNA Kit. cDNA was synthesized using Prime Script RT Reagent (Takara, DRR037A). qPCR was performed on a LightCycler 2.0 using SYBR® Premix Ex Taq™ (Takara, DRR081A). The results were calculated using the 2^-△△Ct^ method[30]. The following primers were used in this study: GAPDH: Forward 5’-GCACCGTCAAGGCTGAGAAC-3’, Reverse 5’-TGGTGAAGACGCCAGTGGA-3’; CD98: Forward 5’-GGGTTCCAGGTTCGGGACATA-3’, Reverse 5’-GGAGGAGTTAGTCCCCGCAAT-3’; CD147: Forward 5’-ACTCCTCACCTGCTCCTTGA-3’, Reverse 5’-GCCTCCATGTTCAGGTTCTC-3’.

### Western blotting and pull-down assay

Cell extracts (30 μg) were separated by sodium dodecyl sulfate-polyacrylamide gel electrophoresis (SDS–PAGE) and transferred onto a polyvinylidene difluoride (PVDF) microporous membrane (Millipore, Boston, MA, USA). The membrane was incubated with primary antibodies against CD147, CD98, integrin, and α-tubulin, according to the manufacturer’s instructions. Horseradish peroxidase-conjugated secondary antibodies (1:5000; Santa Cruz Biotechnology, Santa Cruz, CA) were applied to the membrane and detected using enhanced chemiluminescence reagents (Pierce, Rockford, IL). For the pull-down assay, 10 μg of the HAb18 mAb was first immobilized onto AminoLink Plus Coupling Resin (Pierce kit, Lot: 26149). Then, the bait (CD147-ED) and prey proteins (different amount of CD98-ED) were mixed. The protein mixture and controls (CD98-ED only) were then added to the appropriate resin and incubated. After the resin was washed three times with PBS, the eluted samples were subjected to western blotting.

### Cell-spreading assay

Five thousand cells per cm^2^ were plated onto 1 % Matrigel (BD Bioscience, Franklin Lakes, NJ, USA)-coated glass coverslips. At different time points, the coverslips were removed, and the cells were fixed in PBS with 4 % formaldehyde for 10 min, stained with rhodamine-phalloidin (R415, Invitrogen, USA), and viewed using phase microscopy (Olympus, Tokyo, Japan) [31]. The cells were assessed with ImageJ software (1.47v, National Institutes of Health). The results are the average percent from three independent experiments ± SEM.

### Surface plasmon resonance (SPR) assay

SPR measurements were performed using the ProteOn XPR36 protein interaction array system (Bio-Rad Laboratories) according to a standard procedure. After CD147-ED was immobilized onto the activated GLC sensor chip surface, six different concentrations of CD98-ED (0, 3, 1.5, 0.75, 0.375, 0.1875 μM) were simultaneously injected into the chip for association (180 s) and dissociation (600 s). The binding kinetics was analyzed with ProteOn Manager Version 2.0 software (BioRad).

### Image analysis

#### Immunofluorescence

Cells were cultured in a 24-well plate that had been pre-coated with Matrigel for 3 h. The cells were then fixed with 4 % formaldehyde, permeabilized with 0.2 % Triton X-100 and blocked with 1 % BSA (Beyotime, Shanghai, China) in PBS for 30 min. The cells were incubated with a primary antibody and Dylight488 or Dylight594 labeled secondary antibodies (Life Technologies). The nuclei were counterstained using Vectashield with DAPI (Vector laboratories, Burlingame, CA, USA). The ER was labeled with ER-Tracker™ (E34251, Invitrogen, USA). The samples were visualized with a confocal microscope using Nikon NIS-Elements software (Nikon, Tokyo, Japan).

#### Co-localization analysis

Co-localization data was evaluated in original images, obtained by confocal microscopy. Analysis was performed with Nikon NIS-Elements software (Nikon, Tokyo, Japan).

#### FRET measurements

FRET measurements were performed as previously described [32].

### Flow cytometry analysis

The cells were digested with trypsin, washed twice with PBS and suspended in PBS containing 0.02 % (w/v) sodium azide and 0.1 % (w/v) BSA. After incubation with the primary antibody (HAb 18, CD98, β1 integrin or β1 integrin-A) for 1 h on ice, the cells were washed 3 times and incubated with Dylight488-conjugated secondary antibodies for 45 min at room temperature in the dark and washed twice. Or detached cells were incubated directly with labled antibody (CD147-FITC and CD98-PE) for 1 h on ice and washed twice. The cells were analyzed using a FACS Calibur flow cytometer (Becton Dickinson, San Jose, CA).

### *In vivo* assessment of tumorigenicity

Female BALB/c nude mice (6-weeks-old) were selected from the Laboratory Animal Research Center of FMMU, compliant with the regulations. Animal welfare and experimental procedures were performed according to the NIH Guide for the Care and Use of Laboratory Animals. SMMC7721shNC or SMMC7721shCD98 cells (5 × 10^5^ cells) in 0.2 mL PBS were injected subcutaneously on both sides of the lumbar region. All the mice were observed and examined daily for the formation of nodules at the site of injection. Tumor size was calculated by measuring the dimensions of the tumor mass (average of the two right-angle diameters), and volume was calculated as (4π/3) × (size/2)^3^. The period of observation was one month, after which all mice were euthanized and autopsied. The excised tumors were then weighed.

### Statistical analysis

All statistical tests were performed using SPSS 16 (Chicago, IL, USA). The data are expressed as the mean ± SEM of at least three independent experiments. Comparisons were made using Student’s *t*-test or ANOVA, and a significant difference was defined as *P* < 0.05.

## Results

### CD98 knockdown inhibits SMMC-7721 cell spreading and tumorigenicity *in vitro* and *in vivo*

**Fig. 1:**
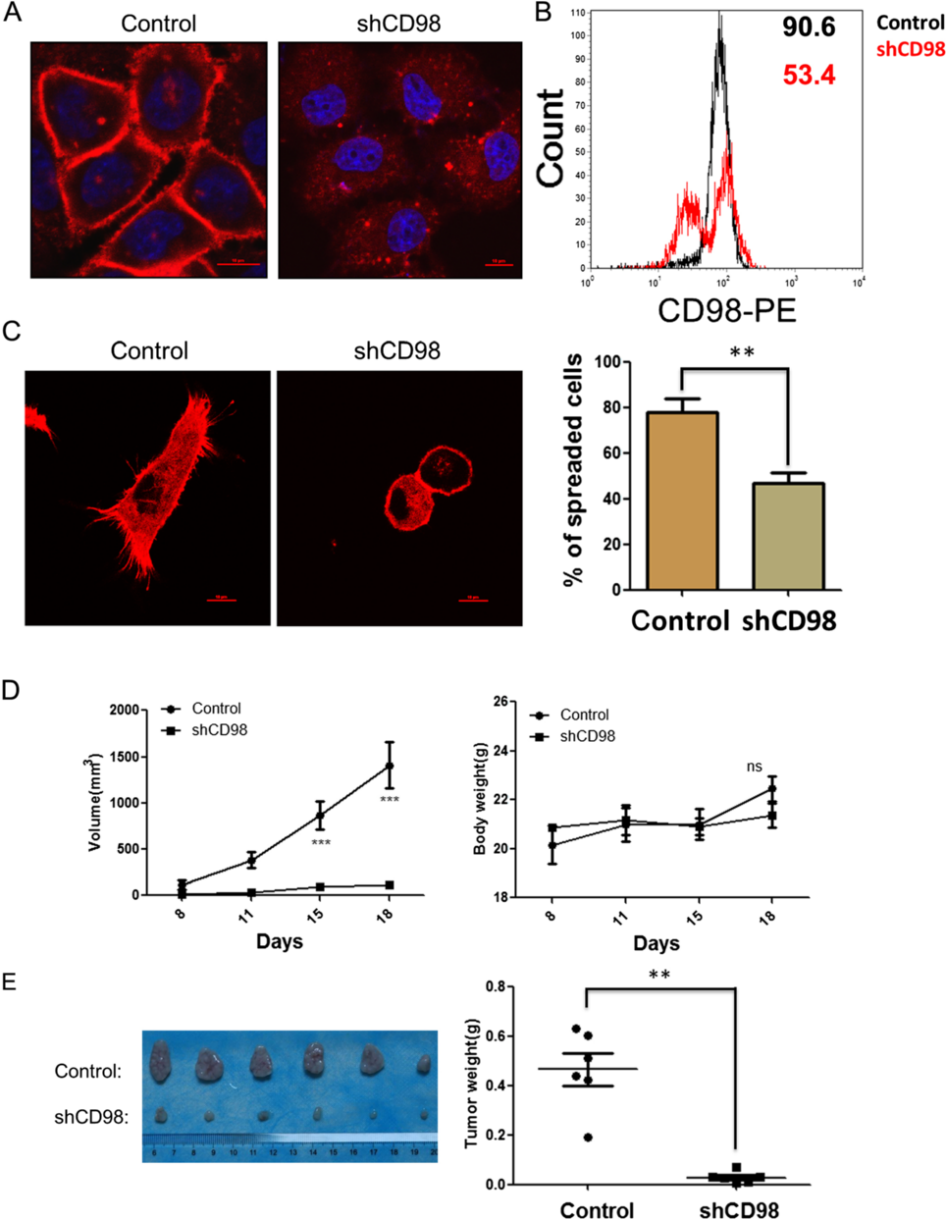
CD98 is involved in the spreading and tumorigenicity of SMMC-7721 cells *in vitro* and *in vivo*. **a** SMMC-7721 cells were transfected with pLKO–SLC3A2(CD98) shRNA (SMMC-7721shCD98) or pLKO–control shRNA plasmid (control) and selected with 5 μg/ml of puromycin for two weeks. The cells (5 × 10^4^ cells) were grown on coverslips for 24 h, fixed and stained with Dylight594-conjugated goat-anti-mouse antibodies (CD98, Red). Representative CD98 immunofluorescence of control and SMMC-7721shCD98 cells are shown in (**a**) DAPI (blue staining). Bar, 10 μm. **b** FCAS analysis of membrane CD98 in control and SMMC-7721shCD98 cells. Growing cells were harvested and incubated with fluorescence-labeled antibody against CD98 (PE) for 1 h. Next, the labeled cells were analyzed using flow cytometry. Mean fluorescence intensities (MFI) of each group was indicated beside the histograms.The data shown are representative of three individual experiments. **c** SMMC-7721 cell spreading assay *in vitro*. Control or SMMC-7721shCD98 cells (5× 10^4^ cells) were cultured on coverslips with 1 % matrigel for 2 h, and then the cells were fixed and stained with rhodamine-phalloidin for visualization (right) and scoring (left). The percentage of spread cells was determined by scoring >100 cells. The data are the mean of the “% of spreaded cells” from three independent experiments ± SED. Bar, 10 μm. ** *P* < 0.01. **d** Tumorigenicity of cells transplanted into nude mice. Control or SMMC-7721shCD98 cells (5 × 10^5^ cells per site) were injected into both sides of 6-weeks-old female nude mice. Tumor volumes were determined at various time points (see Materials and Methods for details). On day 18, the primary tumors were harvested and weighed (**e**). *** *P* < 0.001

To investigate the role of CD98 in HCC cells, a stable CD98 knockdown cell line, SMMC-7721shCD98, was constructed using a shRNA that targeted CD98. As shown in Fig. 1a and 1b, the expression of CD98 was significantly inhibited in SMMC-7721shCD98 cells as analysed by immunofluorescence and FCAS, respectively. SMMC-7721shCD98 cells showed a dramatic reduction in the percentage of spreading cells (43.2 ± 5 %) on Matrigel-coated glass coverslips compared to control cells (78.6 ± 6 %) after 2 h (Fig. 1c). To observe the tumorigenicity *in vivo*, 5 × 10^5^ control or SMMC-7721shCD98 cells were injected into both sides of nude mice. As shown in Fig. 1d, all of the mice in the control group formed nodules at the 8th day. However, only one mouse in the SMMC7721shCD98 group formed nodules at the 11th day. Furthermore, the tumors from the control group grew dramatically, while the body weight of the two groups showed no apparent change. Finally, all of the mice were euthanized, and the tumors were excised and weighed. As shown in Fig. 1d, the weight of the excised tumors in the control group was significantly heavier than those in the SMMC7721shCD98 group (*P* < 0.01). The above results indicated that CD98 can promote cell spreading and tumorigenicity in HCC cells.

### Basigin mediates the membrane localization of CD98 in HCC cells

**Fig. 2:**
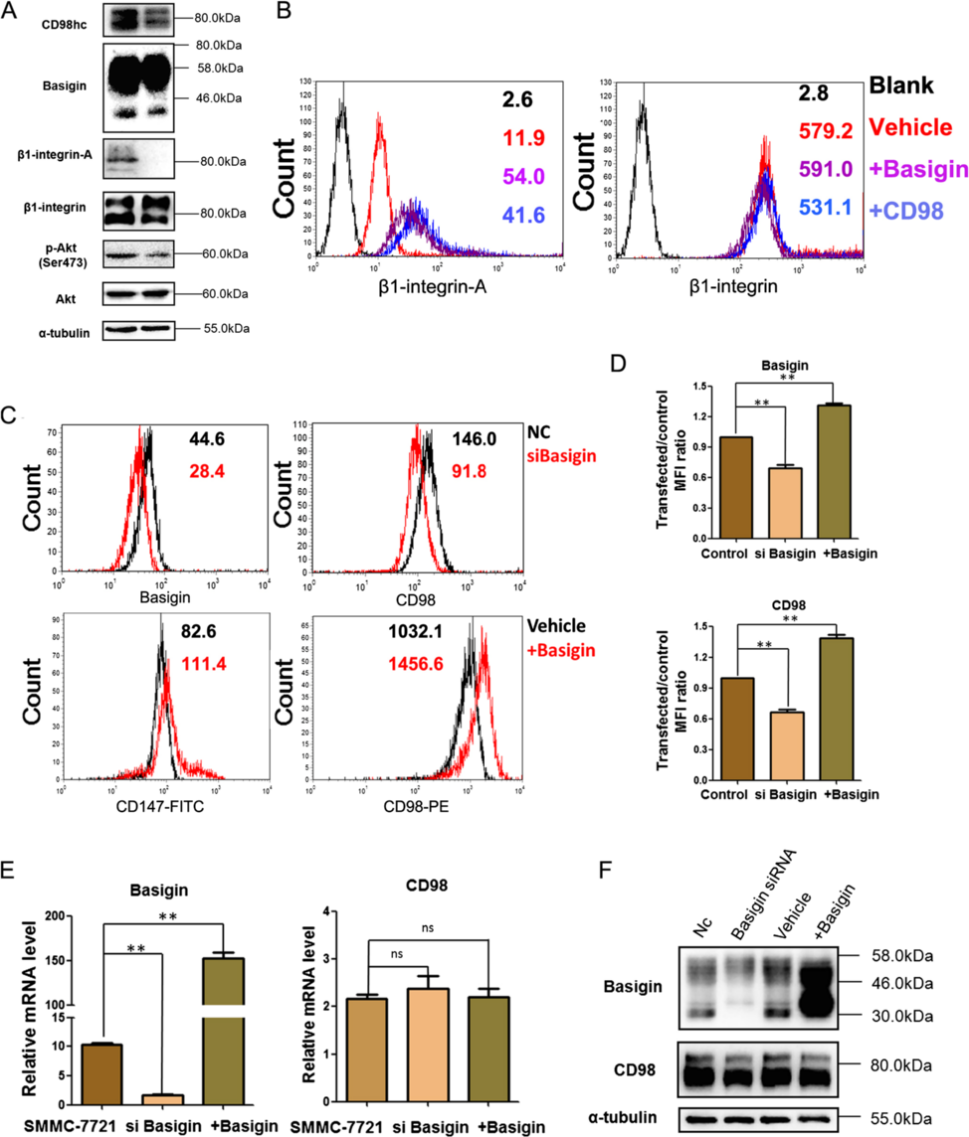
Cell membrane localization of CD98 is mediated by basigin in HCC cells. **a** Changes in the molecular expression patterns were detected in control and SMMC-7721shCD98 cells. Control or SMMC-7721shCD98 cell lysates (30 μg) were subjected to western blot to analyze the expression of basigin, β1-integrin-A, β1-integrin, p-Akt, Akt and α-tubulin (reference control). **b** The membrane expression of β1-integrin-A and β1-integrin were analyzed using flow cytometry after overexpression of basigin or CD98, respectively. Black histograms (Blank) represent staining with isotype control. **c** The membrane localization of CD98 after basigin was knockdown or overexpressed. Mean fluorescence intensities (MFI) of each group was indicated beside the histograms. **d** MFI ratio of transfected cells and control cells. The results shown are representative of three independent experiments. ** *P* < 0.01. **e**The mRNA and protein expression of CD98 in SMMC7721 cells with basigin knockdown or overexpression. After SMMC-7721 cells were transfected with basigin siRNA or pcDNA-3.1-CD147 for 36 h, the mRNA levels of basigin and CD98 were analyzed by RT-PCR. The results shown are representative of three independent experiments. ** *P* < 0.01 (**f**) Analysis of the total protein levels of basigin and CD98 by western blotting. The samples were first transfected as described in part (E) and then subjected to western blotting

The membrane localization of CD98 plays important roles in inducing the malignant phenotypes of cancer cells and its membrane expression was decreased (Fig. 1a and 1b). Here, we found that the activation of β1 integrin and p-Akt were significantly decreased in SMMC7721shCD98 cells compared to the control group (Fig. 2a). Besides CD98 overexpression activated β1 integrin, we also found overexpression of basigin could activate β1 integrin (Fig. 2b and Additional file 1: Supplementary material Fig. S1). Interestingly, when basigin was knocked down, the FACS results showed reduced CD98 membrane localization (Fig. 2c and Fig. 2d, upper pannel), while CD98 at mRNA level (Fig. 2e) or total protein level was not influenced (Fig. 2f). Additionally, cells with ectopic expression of basigin showed a high level of CD98 at the cell membrane (Fig. 2c and Fig. 2d,lower pannel) without influencing mRNA (Fig. 2e) total protein (Fig. 2f). However, when CD98 was knocked down with siRNA, basigin was increased at mRNA level and membrane level (Additional file 1: Supplementary material Fig. S2). These results indicated that the basigin-mediated membrane localization of CD98 was responsible for the activation of downstream integrin-PI3K-Akt signaling, promoting the malignant phenotype of HCC cells.

### CD98 directly interacts with basigin in HCC cells

**Fig. 3:**
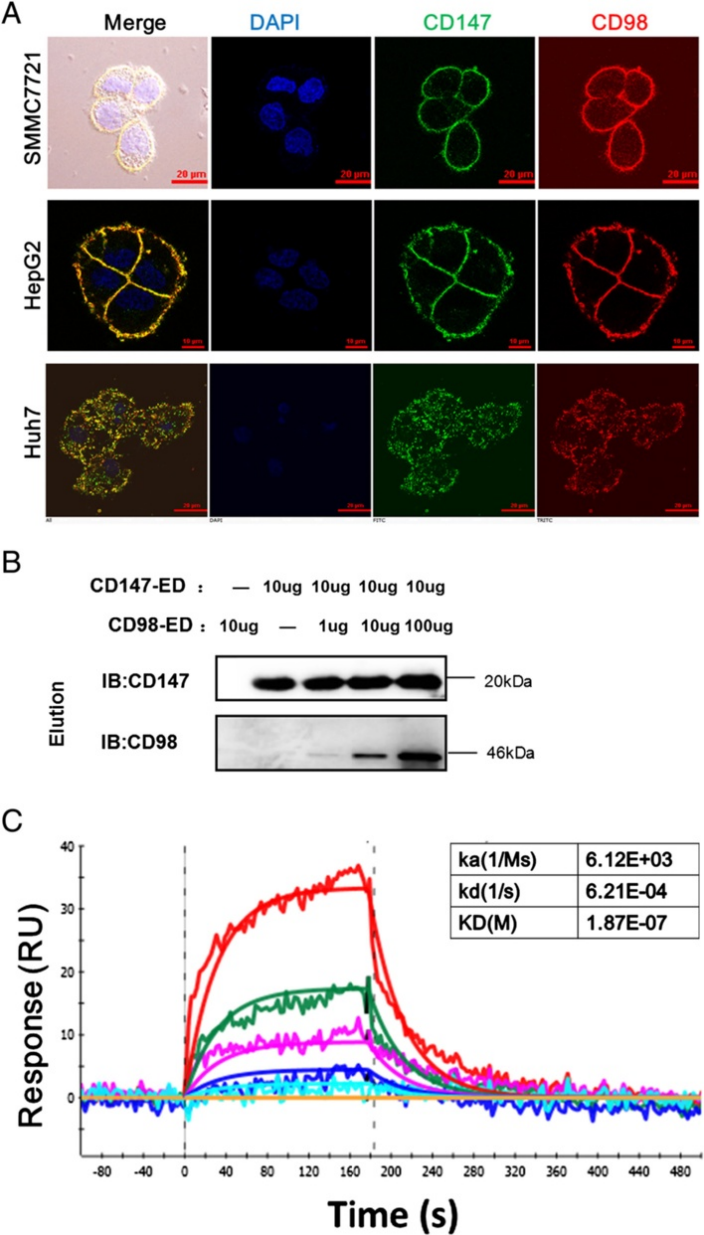
CD98 directly interacts with basigin in HCC cells. **a** Co-localization of basigin and CD98 in SMMC7721, HepG2 and Huh7 HCC cell lines. The cells were grown on coverslips for 24 h, fixed and stained with Dylight488-conjugated goat-anti-rabbit antibodies (basigin, Green) and Dylight594-conjugated goat-anti-mouse antibodies (CD98, Red). Huh7 cells were treated with 0.2 % Triton X-100 after fixation to visualize the intracellular distribution of basigin and CD98. Bar, 10 μm. **b** CD147-ED pulls down CD98-ED *in vitro*. HAb18 (antibody against CD147-ED; 10 μg) was used as a coupling antibody by immobilizing it onto the coupling resin. Then, the indicated amount of the antigen was added and eluted. The collected samples were analyzed by western blotting. **c** Biophysical analysis of the interaction of CD98-ED with CD147-ED using SPR. The indicated concentrations of purified CD98-ED were injected over immobilized CD147-ED, and the biophysical parameters were derived from a 1:1 binding model (red line). RU, response units

To explore how basigin mediates the membrane localization of CD98 in HCC cells, the interaction of these two molecules was analyzed. The immunofluorescence staining results showed that there was a significant colocalization of CD147 with CD98 in HCC cells (SMMC-7721, HepG2 and Huh7; Fig. 3a). The immunoprecipitation results in our previous report showed that CD147 and CD98 could form a complex *in vitro* [21]. To determine whether the two molecules directly interact, we then expressed the extracellular domain of basigin (CD147-ED) [33] and the extracellular domain of CD98 (CD98-ED) in *Escherichia coli* BL21 (DE3) and purified the proteins (Additional file 2: Supplementary material Fig. S3) to further explore their interaction. As shown in Fig. 3b, CD147-ED could pull down CD98-ED in a concentration-dependent manner. Furthermore, surface plasmon resonance (SPR) assays obtained a similar result, in which the SPR data fit well to a one-shot kinetic binding model (chi-squared value 3.73) and revealed an average binding affinity (dissociation equilibrium constant, KD) of approximately 0.187 μM (see table in Fig. 3c). These results were also consistent with our previous report [21] and indicated that basigin directly interacted with CD98.

### Basigin mediates the membrane redistribution of CD98 in HCC cells

**Fig. 4:**
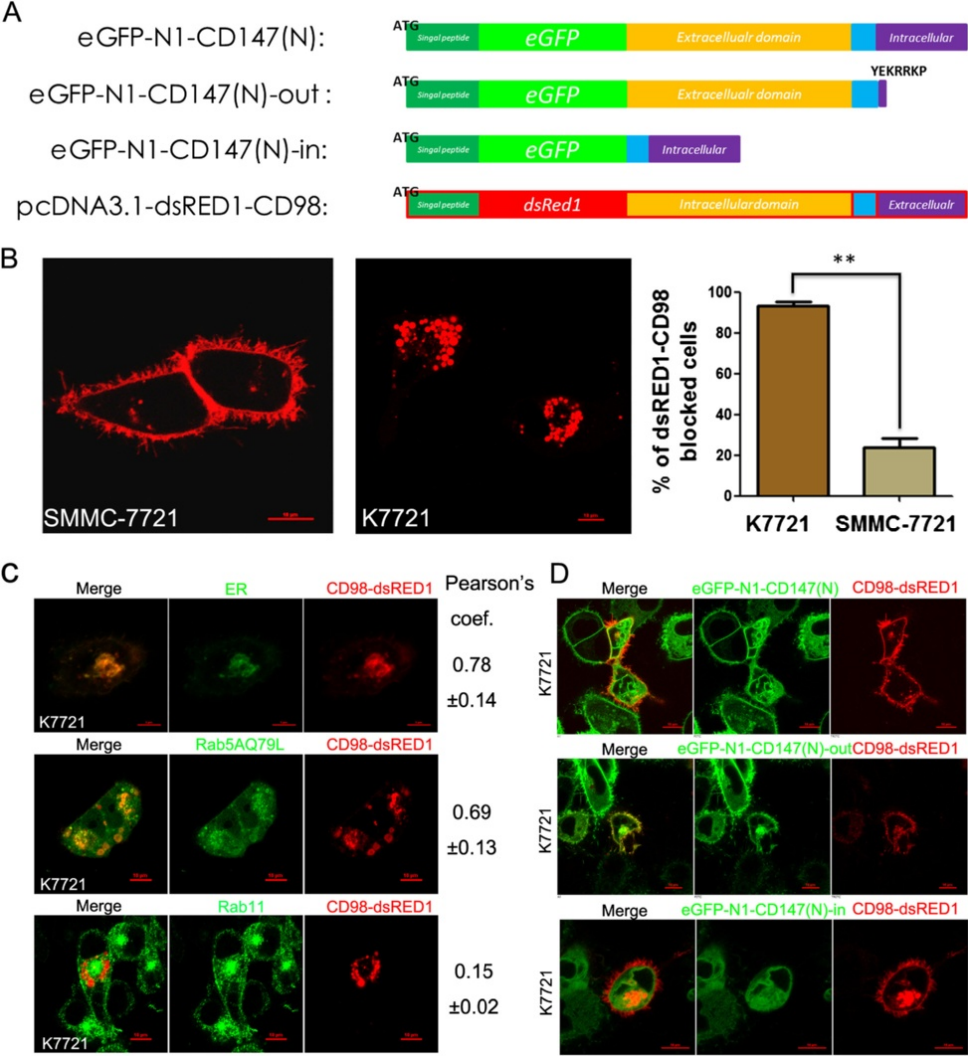
CD98 is retained in the ER and Rab5 positive endosomes of live cancer cells in the absence of basigin. **a** Schematic of the different basigin expression constructs and pcDNA3.1-dsRED1-CD98 (dsRED1-CD98). Note that the sequences encoding eGFP or dsRED1 were inserted into the signal peptide and coding sequence of basigin and CD98, respectively. **b** Representative distribution of overexpressed CD98 (red) in SMMC7721 cells and K7721 cells. SMMC-7721 cells (5 × 10^4^ cells) were cultured on coverslips and transfected with 2 μg of dsRED1-CD98 for 36 h. The distribution of dsRED1-CD98 was visualized with a confocal microscope. The percentage of CD98 (red) blocked cells was determined by scoring > 100 cells. The data are the mean of the “% of dsRED1-CD98 blocked cells” from three independent experiments ± SED. Bar, 10 μm. ** *P* < 0.01. **c** Cytoplasmic distribution of overexpressed CD98 (red) in K7721 cells. After the transfection of dsRED1-CD98 as described in part B, the distribution of the endoplasmic reticulum (ER) was revealed with ER-Tracker^TM^. Bar, 5 μm. EGFP-Rab5A Q79L or GFP-Rab11 (1 μg) was co-transfected with 1 μg of dsRED1-CD98 to visualize the endosomal distribution of cytoplasmically retained CD98. (Pearson’s coefficient is indicated as numerical data on the right of each panel, *n* = 3). Bar, 10 μm. **d** Representative distribution of overexpressed CD98 (red) after co-transfection of different basigin expression constructs. Three different basigin expression constructs (green) were co-transfected with dsRED1-CD98 in K7721 cells for 36 h, and the distribution of overexpressed CD98 (red) was then visualized. Bar, 10 μm

As mentioned above, basigin and CD98 co-localize on the membrane of HCC cells and interact directly *in vitro*. We then overexpressed dsRED1-CD98 in K7721 cells (Additional file 2: Supplementary material Fig. S3) to observe the distribution of CD98 in the absent of basigin. The confocal result showed that dsRED1-CD98 localized to the membrane in the SMMC-7721 cells, while most of the red fluorescent signal was retained in the cytoplasm of the K7721 cells (Fig. 4a and b). Interestingly, we found that dsRED1-CD98 had a well colocalization with the ER (Fig. 4c, upper panel) and Rab5a-positive recycling endosomes in K7721 cells (Fig. 4c, middle panel; GFP-Rab11 in the lower panel is irrelevant). Rab5a endosomes are where internalized membrane proteins (both CIE and CME proteins) converge, and here, we used GFP-Rab5aQ79L to indicate the distribution of this kind of endosome [34–36]. Different portions of basigin fused with eGFP were then co-transfected with dsRED1-CD98 into K7721 cells. The immunofluorescence staining (Fig. 4d, upper and middle panel) showed that both eGFP-N1-CD147(N) and eGFP-N1-CD147(N)-out transfected cells displayed a strong red fluorescent signal at the membrane of the K7721 cells. However, eGFP-N1-CD147(N)-in transfected cells showed red fluorescent signal in the cytoplasm (Fig. 4d, lower panel). The above results indicate that the membrane distribution of CD98 is correlated with the expression of basigin, particularly the extracellular domain, and that basigin may act as a chaperone to promote the membrane localization of CD98.

### Basigin facilitates the early steps of the endocytic recycling process of CD98

**Fig. 5:**
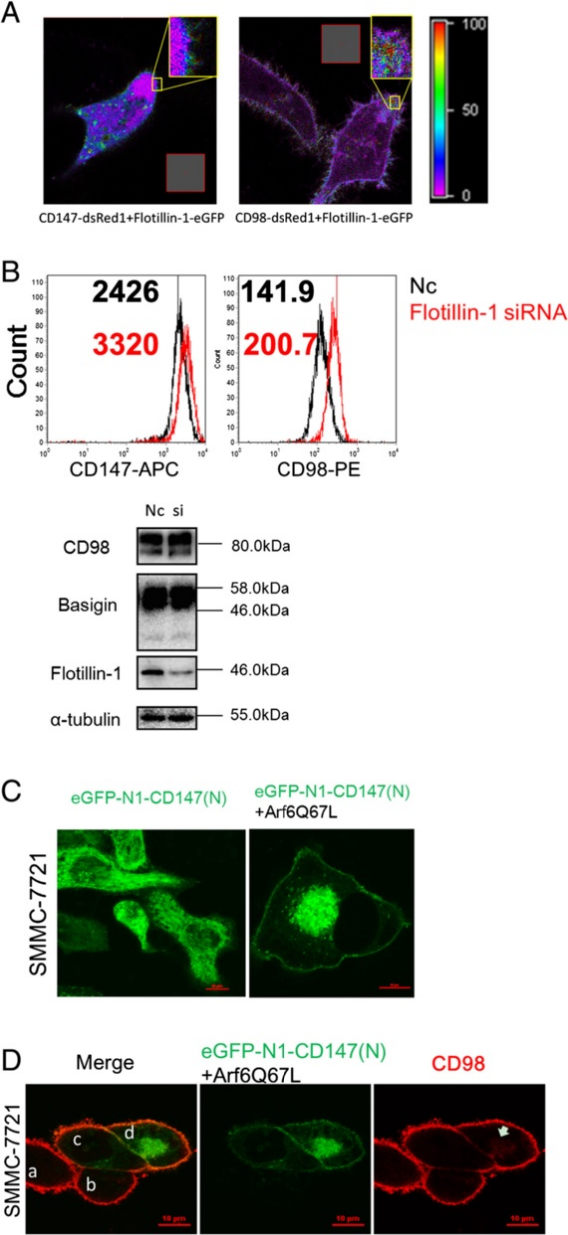
Basigin facilitates the early steps of CD98 recycling. **a** The interaction between basigin and flotillin-1, CD98 and flotillin-1, were analyzed by FRET imaging. Two individual dishes of SMMC-7721 cells were transfected with CD147-dsRed1 or Flotillin-1-eGFP as the acceptor and donor samples, respectively. Two separate dishes were co-transfected with both CD147-dsRed1 and flotillin-1-eGFP as the FRET sample. Using the donor and acceptor samples, CoA and CoB were automatically calculated with FRET software (the grey rectangle area was calculated as background). The FRET ratio is shown on the right. **b** Effects of flotillin-1 knockdown on the membrane localization of basigin and CD98. SMMC-7721 cells were transfected with siRNAs targeting flotillin-1 for 36 h, then the cells were harvested and the membrane localization of basigin and CD98 was analyzed by FACS. The knockdown effect and total protein levels of basigin and CD98 were analyzed by western blotting. **c** Co-transfection of Arf6Q67L for 36 h caused the distribution of overexpressed eGFP-N1-CD147(N) (GFP-CD147) to the vacuolar membranes in SMMC7721 cells. **d** Localization of CD98 in SMMC7721 cells co-expressing GFP-CD147 and Arf6Q67L. SMMC7721 cells co-expressing GFP-CD147 and Arf6Q67L for 36 h were fixed, treated with 0.2 % Triton, and labeled with a primary antibody to CD98 followed by Dylight594-conjugated goat-anti-mouse antibodies. Cell c was transfected with GFP-CD147 only, cell d was transfected with GFP-CD147 and Arf6Q67L (identified by vacuolar membranes), and cells a and b were not transfected and served as a control. Bar, 10 μm

As CD98 was detained in the Rab5a-positive recycling endosomes in the absence of basigin, we examined whether the proteins would be transported together in recycling endosomes. Here, we showed that both basigin and CD98 had a FERT effect with flotillin-1 (Fig. 5a). After knocking down flotillin-1 in SMMC-7721 cells, the membrane localization of basigin and CD98 both increased, as shown in Fig. 5b, while the total amount of basigin and CD98 protein did not change. This result suggests that flotillin-1 may participate in the internalization of basigin and CD98 in SMMC-7721 cells.

After co-expressing GFP-CD147 and Arf6Q67L (constitutively activated form) in SMMC-7721 cells, both basigin and CD98 accumulated in the vacuolar membranes (Fig. 5d and e, cell d, marked with an arrow). Therefore, the expression of Arf6Q67L promoted CIE internalization of both basigin and CD98. Then, the endosomes fused together, causing the accumulation of the cargo proteins in vacuolar-type structures. As to CD98 retaining in the Rab5a-positive recycling endosomes in K7721, the internalized endosomes containing CD98 may have fused with the Rab5a early endosomes, thus blocking further recycling. Together, these results demonstrated that both flotillin-1 and Arf6 influenced the internalization and endocytosis of basigin and CD98 and that basigin may facilitate the early steps of CD98 recycling.

## Discussion

**Fig. 6:**
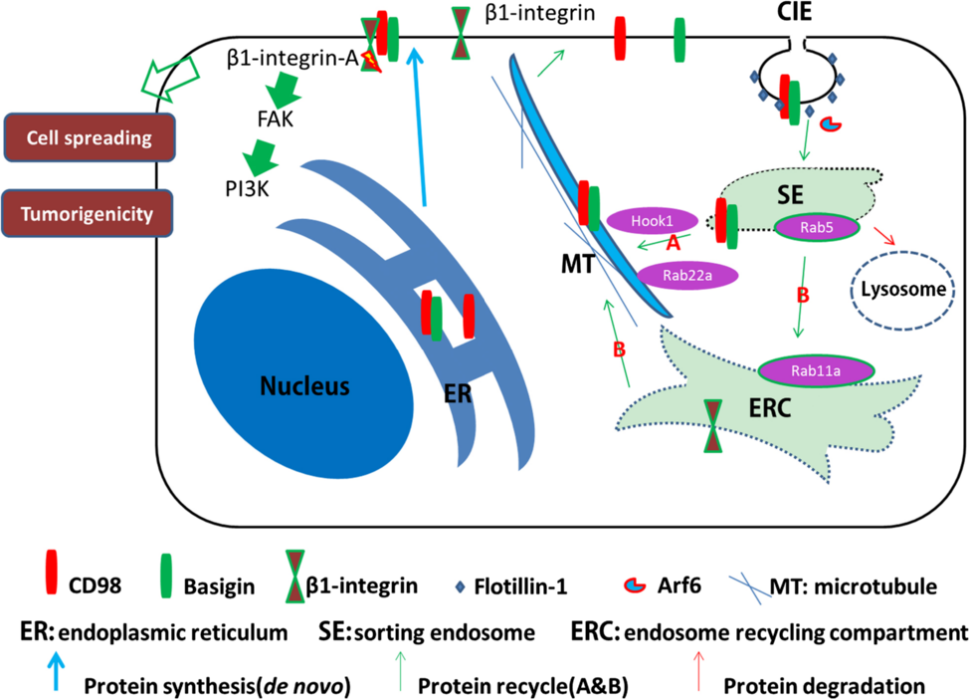
General model for basigin promotes the membrane localization of CD98 and activation of β1-integrin. Basigin could facilitate the membrane localization of newly synthesized CD98 in the ER (blue arrow). Prototypical CIE cargo proteins enter cells through Arf6-positive endocytic vesicles that either fuse with or mature into Rab5a-positive early endosomes (sorting endosomes), where internalized proteins converge and are sorted for recycling (green arrow) or degradation (red arrow). Basigin and CD98 are internalized by a pathway associated with flotillin-1 and the small G protein Arf6. Thereafter, basigin and CD98 could recycle back to the membrane through the fast recycling pathway (green arrow A) mediated by hook1, Rab22a, microtubules and their own cytoplasmic sequences. Other internalized proteins, including integrins, could recycle through the slow recycling pathway (green arrow B). By promoting the membrane redistribution or translocation of CD98, basigin activates β1 integrins and plays a critical role in liver cancer progression

Vesicle recycling plays a critical role in the process of tumor progression through signal transduction and membrane protein redistribution [1, 2]. In this work, we first reported that basigin, a CIE protein, mediates the redistribution and translocation of CD98 to activate β1 integrin and its downstream signaling, which is related to tumor spreading and tumorigenicity in HCC cells. Through direct association with CD98, basigin first facilitated CD98 membrane localization. Second, the two proteins may be transported together efficiently via the fast vesicle recycling pathway. This recycling process avoids the slow recycling pathway through the Rab11-positive endosomes or the default pathway for protein degradation [37] (Fig. 6).

Previously, CD98 was reported to directly interact with and activate β1 integrin and thereby activate components of the downstream signaling pathway, such as FAK, Rac, and Akt [24, 26, 27, 38, 39]. CD98 overexpression is detected in a variety of cancers and is associated with poor clinical prognosis [23, 25]. However, the role of CD98 has not been investigated in liver cancer. In this study, we constructed a stable CD98 knockdown cell line, SMMC-7721shCD98, and found that cell spreading and tumorigenicity were inhibited *in vitro* and *in vivo*. CD98 regulates cell growth and proliferation by being the heavy chain of amino acid transporters LAT1 and LAT2, which are amino acid exchangers and can import essential amino acids (EAA) [40, 41]. CD98 would be detrimental for tumor growth, but at the preliminary stage of tumorigenicity, the role of its cell spreading and adhering to the extracellular matrix is more important. These phenomena observed in our results agree with a defect in integrin signaling. It was reported that β1 integrins are involved in various biological functions, such as cell growth, spreading, migration, metastasis, and immunologic and inflammatory responses. β1 integrins also play an important role in cancer cell signaling, including Rho-GTPases and downstream kinases [42, 43]. In this study, CD98 knockdown significantly inhibited β1 integrin activation and downstream FAK-PI3K signaling, along with a decreased level of p-Akt. Activation of AKT1 (p-Akt)was associated with poor prognosis in cancers like esophageal squamous cell carcinoma [44]. Similarly, ectopic expression of CD98 could activate β1 integrins.

Basigin is a transmembrane glycoprotein and is ubiquitously expressed in cancer tissues, with a significantly higher overall positive rate of 67.76 % than that observed in sarcoma (27.34 %) and normal tissue (5.18 %) [17]. The increased basigin levels were positively correlated with cancer metastasis and progression [45]. As an upstream activator of MMPs, most findings showed that the unregulated basigin increased the secretion and activation of MMPs to promote the invasion and metastasis of cancer cells [20, 46]. Basigin could also activate β1 integrins, but a previous study stressed that basigin binds to the metal ion-dependent adhesion site (MIDAS) motifs of β1 integrins to modulate the malignant properties of hepatoma cells [47]. Basigin was previously shown to interact with CD98, but the precise role of basigin in tumor progression is not clear. Here, we showed that basigin could contribute to the progression of liver cancer by redistributing CD98 to the membrane and activating downstream β1 integrin signaling. Previous studies found that basigin and CD98 formed protein complex by immunoprecipitation *in vitro*, but how they interacted as a complex was not known [21, 22]. Here, we confirmed the direct association of basigin and CD98 and hypothesized that this interaction may participate in the tumorigenicity of HCC cells.

Previous studies have shown that basigin could be a chaperone for MCT1-4 maturation in the ER and its translocation to the membrane [48, 49]. In this study, CD98 was retained in the ER in the absence of basigin, indicating that basigin may function as a chaperone for CD98, or at least the complex containing basigin, MCTs, CD98 and LATs assemble in ER [50]. Moreover, we found that overexpressed CD98 co-localized with Rab5a-positive endosomes in K7721 cells, suggesting that the sorting process was slowed or disrupted. However, transfection of plasmids containing the extracellular domain of basigin could rescue the cytoplasmic arrest of CD98. This suggests that basigin interacts with CD98 and facilitates its recycling in HCC cells.

Previous reports have shown that membrane lipid composition, such as cholesterol, can influence CD98 internalization, but not actin or dynamin [28]. Similar results were also obtained for basigin internalization (data not shown). Specifically, in the present study, we found that flotillin-1, a coat component that mediates CIE, could participate in the internalization of both basigin and CD98. A previous report showed that basigin and CD98 are internalized through Arf6-related CIE in HeLa cells [28]. In this study, we found that both basigin and CD98 are internalized through Arf6-related CIE in HCC cells. After internalization, hook1, Rab22a and microtubules could directly sort basigin and CD98 for recycling, avoiding fusing with EEA1 (early endosome antigen-1)-positive endosomes [10, 11]. Regardless of their localization in vesicles or endosomes, the intracellular domain of CD98 and basigin are exposed to cytoplasm, and the acidic amino acid clusters in the cytoplasmic tails of CD98 and basigin function in the following sorting process to tubular endosomes arranged along microtubules. The coincident localization between CD98 and basigin during the internalization and recycling process, in addition to their direct association, indicates that they are likely to be transported together in cells. Alternative approaches, including live-cell imaging and fluorescence resonance energy transfer (FRET), might track the precise localization of CD147 and CD98 in real time in the future. By binding to each other, basigin and CD98 could use their common sorting machinery (including SNX27 [51], hook1 and Rab22a [10, 11]) more efficiently. Therefore, they could recycle back to the membrane more quickly. Here, our results showed that rapid CD98 recycling was retained in the Rab5a-positive but not Rab11-positive endosomes, in the absence of basigin. Internalized membrane proteins converge in Rab5a endosomes, thereafter proteins are sorted for degradation or recycle. Internalized lipid rafts could recycle through Rab11-positive recycling endosomes (slow recycling pathway) [52–54], while basigin, CD98 and CD44 could recycle back to the membrane through the fast recycling pathway. Here our results further confirmed this fast recycling pathway of basigin and CD98. Meanwhile, it remains to be tested whether CD98 could influence the membrane distribution of basigin by a similar mechanism.

## Conclusions

Basigin facilitates the membrane distribution of CD98 to promote HCC cell spreading and tumorigenicity. Our results supported that endosome recycling emerged as an important signaling platform, capable of controlling the amplitude and duration of oncogenic signals. In this process, the redistribution of CD98 and basigin by vesicle recycling can create markedly altered phenotypes and behaviors in HCC cells. Novel therapies towords HCC might be carried out from the perspective of endosome recycling.

## Additional files

Additional file 1: Fig. S1. Growing SMMC-7721 cells were transfected with pcDNA3.1-CD147 or pCMV5-CD98 for 36 h, respectively. Then cells were collected and applied for western blotting. The data shown are representative of three individual experiments. **Fig. S2.** Growing SMMC-7721 cells were transfected with 50 mM siRNA of CD98 for 36 h. Then cells were detached for FCAS or lysied to extract RNA for RT-PCR. **Fig. S4.** Western blotting was used to identify K7721 cells. SMMC-7721 or K7721 cell lysates (30 μg) were subjected to western blot analysis to detect basigin, CD98 and tubulin expression (reference control) as above. The data shown are representative of three individual experiments. (JPEG 930 kb) Additional file 2: Fig. S3. The cDNA sequence coding the CD98-ED (residues 111–530 of human 4F2hc) was cloned into the pET32a(+)(Invitrogen) with *Nde* I and *Xho* I. The resulting plasmid was transformed into BL21 (DE3) cells (Novagen), and the cells were grown in LB media at 37 °C. Cells with A600 = 0.6 were induced with 0.1 mM isopropyl 1-thio-β-D-galactopyranoside followed by culture for 16 h at 24 °C. Harvested cells were suspended in lysis buffer (20 m M potassiumphosphate, pH 8.0, 500 mM NaCl, 1 mg/ml lysozyme (RocheApplied Science), 1 mM phenylmethylsulfonyl fluoride, aprotinin, pepstatin, andleupeptin) and lysed on a French press at 20,000 p.s.i. The cell lysate was centrifuged twice at 12,000 g for 45 min. The soluble fraction was applied to a His-Trap chelating 5-ml column (AmershamBiosciences Co., Germany), previously charged with Ni^2+^, in an Akta-FPLC. Eluted product was further purified a Superdex 75 gel-filtration column. The antigen was identified through the molecular weight and Western blot. The purification protocols of CD147-ED as described in our previous work [33]. (JPEG 1123 kb)

## Abbreviations

HCC: Human hepatocellular carcinoma
RTKs: Receptor tyrosine kinases
CME: Clathrin-mediated endocytosis
CIE: Clathrin-independent endocytosis
EMMPRIN: Extracellular matrix metalloproteinase inducer
ER: Endoplasmic reticulum
EAA: Essential amino acids
MIDAS: Metal ion-dependent adhesion site
EEA1: Early endosome antigen-1
FRET: Fluorescence resonance energy transfer
