# Supplementary figures and images for: Basigin-mediated redistribution of CD98 promotes cell spreading and tumorigenicity in hepatocellular carcinoma
Source: J Exp Clin Cancer Res. 2015 Oct 6;34:110. doi: 10.1186/s13046-015-0226-6 (PMC4594993; doi:10.1186/s13046-015-0226-6)

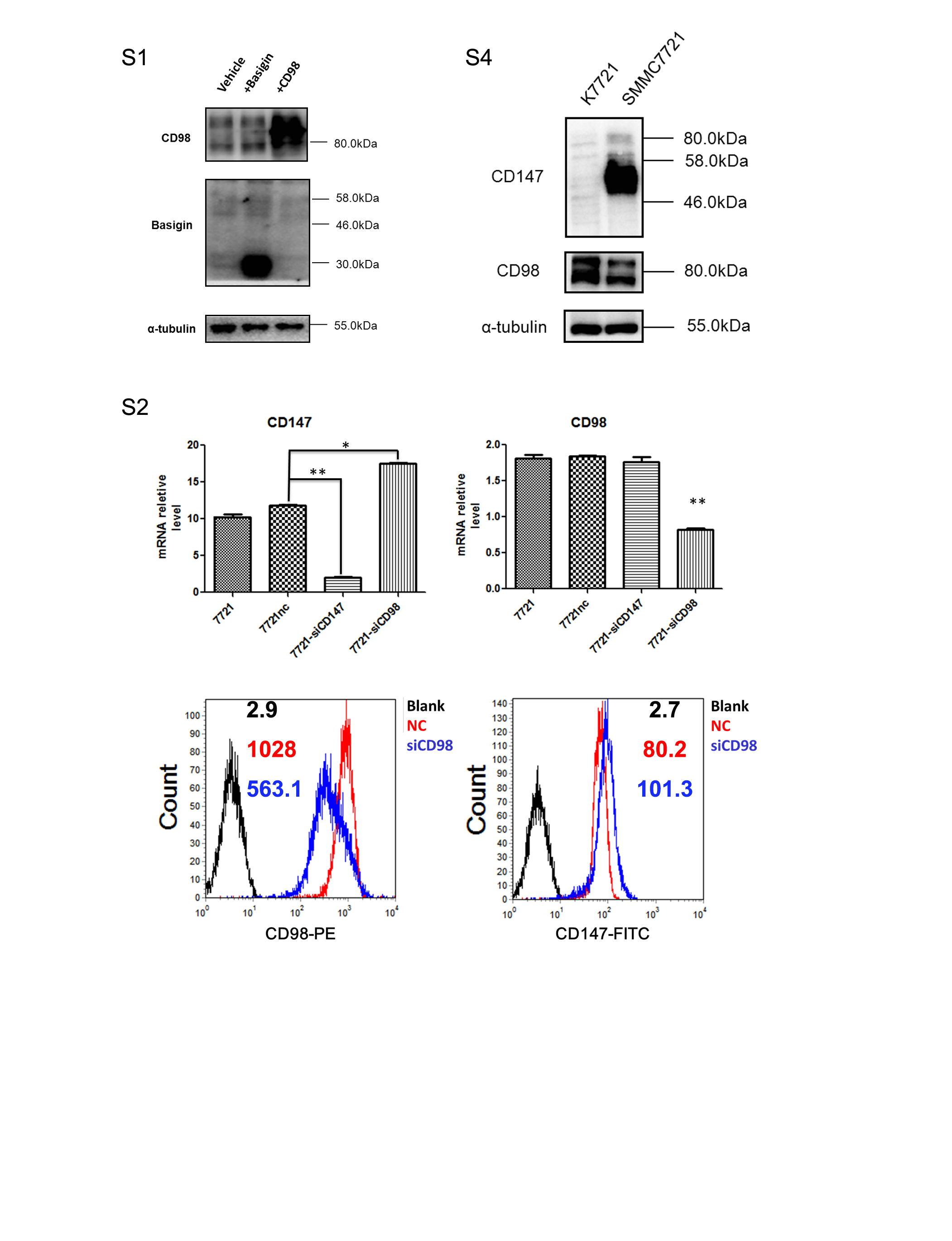

Supplement: Additional file 1: Fig. S1. — Growing SMMC-7721 cells were transfected with pcDNA3.1-CD147 or pCMV5-CD98 for 36 h, respectively. Then cells were collected and applied for western blotting. The data shown are representative of three individual experiments. Fig. S2. Growing SMMC-7721 cells were transfected with 50 mM siRNA of CD98 for 36 h. Then cells were detached for FCAS or lysied to extract RNA for RT-PCR. Fig. S4. Western blotting was used to identify K7721 cells. SMMC-7721 or K7721 cell lysates (30 μg) were subjected to western blot analysis to detect basigin, CD98 and tubulin expression (reference control) as above. The data shown are representative of three individual experiments. (JPEG 930 kb) [file 13046_2015_226_MOESM1_ESM.jpg]
